# Prevalence, Associated Factors and Consequences of Unwanted Pregnancy in Iran

**DOI:** 10.18502/ijph.v49i8.3897

**Published:** 2020-08

**Authors:** Mohammad Esmaeil MOTLAGH, Seiyed Davoud NASROLLAHPOUR SHIRVANI, Zahra HASSANZADEH-ROSTAMI, Farahnaz TORKESTANI, Seyed-Mozaffar RABIEE, Hassan ASHRAFIAN AMIRI, Laleh RADPOOYAN

**Affiliations:** 1. Department of Pediatrics, School of Medicine, Ahvaz Jundishapur University of Medical Science, Ahvaz, Iran; 2. Social Determinants of Health Research Center, Health Research Institute, Babol University of Medical Science, Babol, Iran; 3. Nutrition Research Center, School of Nutrition and Food Sciences, Shiraz University of Medical Sciences, Shiraz, Iran; 4. Department of Obstetrics and Gynecology, School of Medicine, Shahed University, Tehran, Iran; 5. Department of Anesthesiology and Intensive Care, Babol University of Medical Sciences, Babol, Iran; 6. Babol University of Medical Sciences, Babol, Iran; 7. Department of Health, Ministry of Health and Medical Education, Tehran, Iran

**Keywords:** Unwanted pregnancy, Risk factors, Consequences, Prevalence

## Abstract

**Background:**

Unwanted pregnancy is a type of unplanned pregnancy that can endanger health of mother and child. This study aimed to determine the prevalence of unwanted pregnancy and its associated factors and consequences in Iran.

**Methods:**

This cross-sectional study was conducted in regions with low, moderate and high risk of maternal death. Two provinces were randomly selected in each region and 24 public health centers in each province during 2007–2012. Thereafter, 15–20 mothers, received at least one session of pregnancy care, were selected from each healthcare center. Data were gathered from both health records and interview with the mothers.

**Results:**

Of 2714 participants, 86.4% and 13.6% had respectively wanted and unwanted pregnancies. The underlying factors of unwanted pregnancy were determined as low distance with previous and next pregnancy, economic problems and have enough children. Moreover, there were significant relationships between unwanted pregnancy and place of residence, mother’s age and education, father’s education, pre-pregnancy care and number of previous pregnancies and children. There were also significant association between unwanted pregnancy and pregnancy care, anemia, exposure to risk factors and disease, intake of folic acid and iron, domestic violence, bitter memories and men’s participation.

**Conclusion:**

Although the prevalence of unwanted pregnancy has had a significant decrease in Iran, these mothers still require a higher level of educational, counseling and supportive services due to their low access to pregnancy care services and high exposure to associated risk factors.

## Introduction

A planned and purposeful pregnancy could improve both mother and newborn’s health (1). Unwanted pregnancy occurs without any prior planning, and at least one parent was not ready or willing to accept it (2). This type of pregnancy is a global problem that occurs in all countries regardless of their socio-economic status and definitely affects the women, their families, and societies (3). Although the global rate of unwanted pregnancy dropped by 17%, from 1995 to 2008, 41% of all pregnancies (4 out of every 10 case) are still happen unwanted (4). Each year, fifty million women decide to abortion due to unwanted pregnancy, and 42 million of them do this, which approximately half of abortions (20 millions) occur unsafely (4).

Each women need acceptance of pregnancy and preparation for it, to be healthy during pregnancy, childbirth, and after that, care of newborn. However, unplanned pregnancy is accompanied by fear, worries and anxiety during pregnancy. Women with unwanted pregnancy are more susceptible to hypertension, miscarriage, preterm labor, low birth weight and reduced marital satisfaction comparing to women with planned pregnancy, due to unpleasant feelings and emotions during pregnancy (5, 6). Furthermore, a mother with unwanted pregnancy don’t regularly go to healthcare center, and don’t take necessary cares for her and her fetus. Therefore, some probable disorders in pregnancy such as preeclampsia and eclampsia, seriously threaten the health of mother and fetus, could not be timely diagnosed and prevented (5, 6).

Previous studies all around the world have presented numerous reasons for occurrence of unwanted pregnancy and the subsequent induced abortion. Some of these reasons include having enough children, not being ready to take care of children, concerns about the mother and fetus’s health, incapability to continue education whilst having a child, poor marital relationship and intentions of divorce, financial problems, feelings of loneliness, insecurity toward the future, and being pressured into abortion by the society, family members such as the husband or even healthcare service providers (7).

Various studies have been conducted to assess prevalence, causes and consequences of unwanted pregnancy in Iran, reported various findings. The rate of unwanted pregnancy was reduced from 32% to 21%, during 2000 to 2009 (8). However, despite this significant decrease in the rate of unwanted pregnancy, it still have a high prevalence in some Iranian regions. Age, number of previous pregnancies, education, economic status, spouse’s occupation, birth control methods and marital relationship have been previously identified as underlying factors of unwanted pregnancy in Iran (9).

Other than abortion, stillbirth, low birth weight and maternal death, which identified as consequences of unwanted pregnancy (10,11), mothers with unwanted pregnancy were in worse condition of physical performance, role in the family, physical pain, overall health, vitality, social function, emotional and mental health compared to mothers with wanted pregnancy. Mental health and vitality reduced in women with unwanted pregnancy 9.2 times and 5.2 times more than women with planned pregnancy, respectively (12). Considering the high prevalence of unwanted pregnancy and its consequences, some solution tips were proposed to prevent unwanted pregnancy, including provision of education to parents, considering the appropriate age for pregnancy, using birth control methods, participation of men in family planning programs, and improving the quality of family planning services (13).

Given the high prevalence of unwanted pregnancy and its probable worse outcomes, the policymakers and officials of the Iranian Ministry of Health and Medical Education, who compiled the National Program of Maternal Health, considered unwanted pregnancy as one of the 17 risk factors listed in the pregnancy care forms, used in primary healthcare units of healthcare network system. Healthcare service providers must determine the pregnancy status at the first visit, and if the pregnancy was unwanted, record this information as a potential risk factor. In this regard, these healthcare units need to gradually prepare the mothers to accept the pregnancy and childbirth by provision of necessary education, so that they would cooperate with the preventive measures and receive the required pregnancy care.

The present study was aimed to determine the prevalence of unwanted pregnancy in Iran and its associated factors and outcomes.

## Materials and Methods

### Study Population

This was a cross-sectional study conducted in the fall of 2015. The study subjects were selected from Iranian women within the ages of fertility. Based on a national study of maternal death among Iranian women during 2007–2012, which classified the Iranian provinces into three categories of low-risk, average-risk and high-risk of maternal death (9), we randomly selected six provinces including two low-risk provinces with less than 15 maternal deaths per 100,000 live births (Chaharmahal & Bakhtiari and Hamadan), two average-risk provinces with 15–25 deaths per 100,000 births (West Azerbaijan and Razavi Khorasan) and two high-risk provinces with higher than 25 deaths (Golestan and Hormozgan). Considering the sample size of more than 400 mothers per each province. Then 24 urban and rural public healthcare centers were randomly selected in each province, proportional to the geographical distribution of population. And, 15–20 mothers, received at least one session of pregnancy care, were selected from each healthcare center. We collected data from health records of women who covered by each center during pregnancy and had went into labor more than 2 months prior to the study.

The study was approved by Ethics Committee of Ahvaz Jundishahpour University of Medical Science, by ID number of AJUMS.REC.1393.119. Participants were informed about study and verbal consent were obtained.

### Data Collection Tools

We used a checklist designed based on the structure of pregnancy care forms used in the mothers’ health records, which including region under study, resident area (urban/rural), urban population of the cities under study, mother’s age, education and occupation, father’s age, education and occupation, pre-pregnancy care, number of pregnancies, miscarriages, stillbirths and live births, exposure to risk factors and disease during pregnancy, time of the first pregnancy care visit, number of pre- and postnatal care visits, mother’s BMI, hemoglobin test results from the first and third trimesters and number of folic acid and iron intakes. Moreover, we used a questionnaire to interview mothers and collect data of type of housing (apartment/non-apartment), residential area (private/rental), personal or family vehicle ownership status, distance between the place of residence and the care center (on foot), pregnancy occurrence status (wanted or unwanted from the perspective of both parents), number of mother’s marriages, kinship between parents, use of birth control methods and their types, reasons for not wanting the pregnancy, exposure to domestic violence (physical and psychological), having bitter and unpleasant memories associated with pregnancy, childbirth and the postpartum period.

There were also 13 questions about participation of men in prenatal, childbirth and postpartum care processes, including attending at home, providing the necessary amenities, help in housekeeping, ask his sister or mother for auxiliary services, providing appropriate foods, avoid intercourse as needed, reminding use of drug and/or supplements, accompany his wife to go to health care units, accompany his wife to participate in educational class, perceive the probable weakness in pregnancy and labor, intend and advise to vaginal childbirth, presence during childbirth, and cooperation in baby care. These items were scored by Likert scale from 1 to 5 score, based on very low to very high.

### Statistical Tests

Data were analyzed using SPSS Statistics (Chicago, IL, USA, Ver. 18.0) at the significance level of α<0.05. The T-test was used to determine the relationships between means, and Chi-square test was employed for nominal qualitative variables. The association between the potential risk factors and unwanted pregnancy was assessed using the logistic regression model. Logistic regression was used to analyze factors including, pregnancy condition (wanted/unwanted), geographical region (low and moderate risk/high risk of maternal death), number of previous pregnancy (0–2 times/more than 2 times), number of children (0–2/more than 2), history of pre-pregnancy care (yes/no), mother’s education (illiterate – middle school diploma/high school diploma – higher education), Husband’s education (illiterate – middle school diploma/high school diploma – higher education), and mother’s age (lower than 30/higher than 30).

## Results

Among the 2714 mothers interviewed and evaluated based on the prenatal and postpartum history documented in their health records, 2345 had wanted pregnancy (86.4%) and 369 had unwanted pregnancy (13.6%). We found in 1.8%, 1.3%, and 10.5% of unwanted pregnancies, respectively mothers, fathers, and both parents unwanted the pregnancy. Among unwanted pregnancies, 270 couples (20.9%) had used one birth control method, which had been natural or sporadic in 35.6% of the cases. Moreover, 88 mothers (23.8%) reported that lack of enough time between this pregnancy and the previous one was their main reason for not wanting the pregnancy. Moreover, 77 individuals (20.9%) pointed to economic problems as their reason and 69 (18.7%) stated that they already had enough children. Table 1 presents the underlying factors of unwanted pregnancy.

**Table 1:** Factors associated with unwanted pregnancy in Iran

***Variable***		***Wanted pregnancy***	***Unwanted pregnancy***	***Total***	***P- Value***
Regions under Study	Low Risk of Maternal Death	709(82.6)	149(17.4)	858	<0.001
	Average Risk of Maternal Death	778(88.3)	103(11.7)	881	
	High Risk of Maternal Death	858(88.0)	117(12.0)	975	
Mother’s Age	Under 20	153(88.4)	20(11.6)	173	0.003
	20 – 30	1473(88.0)	201(12.0)	1674	
	Over 30	686(83.2)	139(16.8)	825	
Mother’s Education	Illiterate – Middle School Diploma	978(85.5)	166(14.5)	1144	0.04
	High School Diploma – Higher Education	1295(87.9)	179(12.1)	1475	
Husband’s Education	Illiterate – Middle School Diploma	1107(85.2)	192(14.8)	1299	0.01
	High School Diploma – Higher Education	1169(88.2)	157(11.8)	1326	
Number of Previous Pregnancies	No Previous Pregnancies	827(93.7)	56(6.3)	883	<0.001
	Once	780(86.5)	122(13.5)	902	
	Twice	416(80.5)	101(19.5)	517	
	Three times	170(76.2)	53(23.8)	223	
	Four times & more	76(76.0)	24(24.0)	100	
Number of Children	No Children	827(93.6)	57(6.4)	884	<0.001
	One Child	884(87.1)	131(12.9)	1015	
	Two Children	353(75.3)	116(24.7)	469	
	Three Children	80(69.0)	36(31.0)	116	
	Four Children & more	25(65.8)	13(34.2)	38	
History of Pre-pregnancy Care	No History of Care	1065(81.8)	237(18.2)	1302	0.001
	One Period of Care	1098(90.3)	118(9.7)	1216	
	Two Periods & higher	182(92.9)	14(7.1)	196	

* *P*-value was resulted from Pearson chi-square Test

Unwanted pregnancy was not statistically associated to resident area, urban population, type of housing (apartment/non-apartment), residential ownership status (private/rental), personal or family vehicle ownership status, distance between the place of residence and the healthcare center, number of mother’s marriages, kinship between parents, mother’s occupation, father’s occupation, father’s age, history of miscarriage, history of stillbirth and mother’s BMI (*P*>0.05).

Table 2 indicates significant association between prevalence of unwanted pregnancy and some demographic variables, as identified via the Chi-square test. Furthermore, Binary Logistic Regression test showed geographical region, number of children, and pre-pregnancy care were significantly related to unwanted pregnancy.

**Table 2:** Relationships between demographic variables and unwanted pregnancy in Iran

***Variable***	***OR***	***95% CI***	***P value***
Geographical regions based on maternal death rate (low-risk / average-risk / high-risk^*^)	1.705	1.331–2.185	<0.001
Number of previous pregnancies (0–2 times / more than 2 times^*^)	1.004	0.696–1.449	0.98
Number of children (0–2 children/more than 2 children^*^)	0.285	0.208–0.391	<0.001
Pre-pregnancy care (no history / once and more^*^)	2.124	1.658–2.721	<0.001
Mother’s education (illiterate-middle school diploma / high school diploma-higher education ^*^)	0.881	0.671–1.159	0.36
Husband’s education (illiterate-middle school diploma / high school diploma-higher education ^*^)	1.119	0.858–1.460	0.40
Mother’s age (under 30 / 30 and over^*^)	1.167	0.876–1.555	0.29

* Reference category

There was no significant relationship between the incidence of unwanted pregnancy and hemoglobin levels in the third trimester (anemia and no anemia) (*P*=0.05). Moreover, there was a significant association between the prevalence of unwanted pregnancy and the mean score of men’s participation in prenatal and postpartum care. Out of a total score of 65, men with wanted pregnancy got a score of 51.3±9.5 and men with unwanted pregnancy received a mean score of 45.1±11.3 (*P*=0.001).

Table 3 shows factors statistically identified as unwanted pregnancy outcomes, using chi-square test. Based on Table 3, the 11 outcomes identified to be significantly affected by unwanted pregnancy were each individually evaluated along with the seven variables discussed in Table 1 using an adjusted version of Binary Logistic Regression. Results just showed significant relationships between the prevalence of unwanted pregnancy and domestic violence and bitter and unpleasant memories during pregnancy. Thereby, the odds of physical (OR; 0.47, 95% CI; 0.31, 0.70), psychological (OR; 0.51, 95% CI; 0.38, 0.67) domestic violence, and having bitter or unpleasant memories (OR; 0.74, 95% CI; 0.55, 1.00) in the pregnancy period was higher in women with unwanted pregnancy.

**Table 3:** The relationship between unwanted pregnancy and certain outcomes

***Variable***		***Wanted pregnancy***	***Unwanted pregnancy***	***Total***	P- Value^*^
Commencement of the first session of pregnancy care	Within the first 4 months of pregnancy	1732(88.4)	228(11.6)	1960	<0.001
	4 months into pregnancy & later	148(79.1)	39(20.9)	187	
Adequacy of the pregnancy care	Inadequate	570(82.3)	123(17.7)	693	<0.001
	Adequate	1665(87.8)	232(12.2)	1897	
Hemoglobin levels in the first trimester	<11 mg/d (anemia)	173(82.0)	38(18.0)	211	0.02
	=> 11 mg/d (lack of anemia)	2037(87.1)	301(12.9)	2338	
Exposure to risk factors or disease during pregnancy	No exposure to risk factors or disease	815(92.2)	69(7.8)	884	<0.001
	Exposure to at least one risk factor or disease	1530(83.6)	300(16.4)	1830	
Folic acid intake	1–5 times	1670(86.4)	265(13.6)	1944	0.02
	6–10 times	403(90.0)	45(10.0)	448	
Iron intake	1–5 times	1302(84.9)	232(15.1)	1534	0.004
	6–10 times	912(88.5)	118(11.5)	1030	
Number of postpartum care visits	No postpartum care visits	135(79.9)	34(20.1)	169	0.01
	Once	257(84.5)	47(15.5)	304	
	Twice	867(86.0)	141(14.0)	1008	
	Three times	1086(88.1)	147(11.9)	1233	
Physical domestic violence	No exposure to domestic violence	2164(87.2)	318(12.8)	2482	<0.001
	Exposure to domestic violence	166(77.2)	49(22.8)	215	
Psychological domestic violence	No exposure to domestic violence	1772(88.4)	233(11.6)	2005	<0.001
	Exposure to domestic violence	560(80.6)	135(19.4)	695	
Bitter and unpleasant memories during pregnancy	Lack of bitter memories	749(89.2)	91(10.8)	840	0.002
	Existence of bitter memories	1541(85.0)	271(15.0)	1812	
Bitter and unpleasant memories in the postpartum period	Lack of bitter memories	660(88.2)	88(11.8)	748	0.04
	Existence of bitter memories	1633(85.8)	271(14.2)	1904	

* *P*-value was resulted from Pearson chi-square Test

## Discussion

Approximately one out of every 7–8 pregnancies in Iran were unwanted. We found living in regions with low risk of maternal death, having more than two children, and having no history of pre-pregnancy care were main determinants of unwanted pregnancy.

This rate of unwanted pregnancy is much lower comparing to reports from previous studies in Iran with smaller samples or even foreign studies in the field. In a meta-analysis reviewing 49 articles with a total sample size of 43061 cases in Iran, the incidence of unwanted pregnancy was calculated as 30.6% (13). The rate of unwanted pregnancy was reported as 24% and 36.5% in Ethiopia (14, 15). This decreased rate of unwanted pregnancy in Iran could have a number of explanations. First, it can indicate an increased level of awareness among Iranian couples within fertile ages, who not only have a better knowledge of safe birth control methods and the failure rate of natural or sporadic methods but have acquired a deeper understanding of the risks associated with unwanted pregnancy, including the possibility of induced abortion.

Secondly, another reason for this lower rate of unwanted pregnancy, compared to foreign studies in particular, could be the fact that only legitimate pregnancy cases visited the healthcare centers in this study. Therefore, the firm family values in Iran and the rarity of illegitimate relationships among married women might explain the lower incidence of unwanted pregnancy in this country.

Findings from the present research revealed that unwanted pregnancy has significantly different rates in different geographical regions. This difference could be associated with differences in the parents’ level of education or could even be indirectly linked to the different cultures and perspectives.

Results showed having more than 2 children is related to unwanted pregnancy. Consistently, a national study in Ethiopia reported higher rate of unwanted pregnancy, when the children number were increased (16). Moreover, other studies also revealed unwanted pregnancy more happened in women who did not want more children, at period of conception (17, 18). Not wanting more children may be due to low distance with previous births, inappropriate economic situation, believing to have enough children, and age of mother, that each factor could depend on age of marriage, education level, resident area, religious, ethnicity, etc. (18–20).

This study suggested that occurrence of unwanted pregnancy was significantly associated with both mother and father’s education (with higher rates among illiterate or less educated individuals), which is consistent with reports from other researchers, such as Ghana and Russia (21, 22). A higher level of education would encourage people to attain more health information, and thereby, be more willing toward behavioral change and modification.

The current research shows that occurrence of unwanted pregnancy is associated with mother’s age. In this regard, women over 30 yr old are more prone to unwanted pregnancy. This finding is not consistent with another results, which suggested the higher prevalence of unintended pregnancy among younger women in Kenya (14). One reason for this inconsistency might be the fact that only married women were considered in the present study. Thus, again the firm family values and occurrence of pregnancy within familial frameworks can explain the lower rate of unwanted pregnancy among Iranian youth. The higher prevalence of unwanted pregnancy in the 30-and-over age group can indicate the concerns that couples have toward the potential consequences of pregnancy in older ages. It could also be due to the fact that older individuals usually already have the desired number of children are not interested in having any more.

This research indicated that women with unwanted pregnancy are more exposed to domestic violence (physical and psychological) and have more bitter and unpleasant memories of their pregnancy period. A study as well has confirmed the higher prevalence of domestic violence in women with unwanted pregnancy (23). To explain this finding, men usually blame their wives for the occurrence of unwanted pregnancy and have the delusion that birth control is the woman’s responsibility. This finding can be an alarm to healthcare service providers that they need to more seriously and alertly than ever identify the mothers who don’t have the intention or preconditions of a new pregnancy, and try to minimize the rate of unwanted pregnancies by provision of effective counseling and education toward better participation of men.

Our findings also revealed that unwanted pregnancy could act as an inhibiting barrier and deprive the mothers of receiving pregnancy care in a timely fashion, cause them to receive and consume less drugs and supplements and even make them more prone than others to the risk factors and diseases associated with pregnancy. Women with unplanned pregnancy received a less amount of pregnancy care (23). Mothers with unintended pregnancy were less successful in breast-feeding and social interactions and had a generally lower self-esteem (24).

Another finding involved the lower level of men’s participation in prenatal and postpartum care in cases of unwanted pregnancy. Similar to the subject of domestic violence, this finding shows that unwanted pregnancy can significantly reduce the husband’s enthusiasm, which would have a more impact on women when the man is the reluctant party.

This study was limited to include mothers who covered just by private sectors, although mostly covered by public sectors in rural area. Furthermore, we limited to include mothers who didn’t give health care at any time, had miscarriage, and have been dead during pregnancy and/or labor.

## Conclusion

Although there has been a significant decrease in the prevalence of unwanted pregnancy, women with unwanted pregnancy still require more help than others due to their lower access to pregnancy care services and higher exposure to pregnancy risk factors, regardless of how large their population is. Thus, healthcare officials need to take measures to seriously and actively identify these mothers in order to provide them with better and faster care and put them in a priority for educational, counseling and supportive services.

## Ethical considerations

Ethical issues (Including plagiarism, informed consent, misconduct, data fabrication and/or falsification, double publication and/or submission, redundancy, etc.) have been completely observed by the authors.
